# Is ‘number sense’ a sense?

**DOI:** 10.7554/eLife.108701

**Published:** 2025-09-09

**Authors:** Toshiya Matsushima

**Affiliations:** 1 Department of Biology, Faculty of Science, Hokkaido University Sapporo Japan

**Keywords:** spatial-numerical association, mental number line, number sense, numerical cognition, brain lateralization, hemispheric specialization, Chicken

## Abstract

Experiments on domestic chicks shed light on the links between brain lateralization and the left-to-right mental number line.

**Related research article** Rugani R, Macchinizzi M, Zhang Y, Regolin L. 2025. Prenatal light exposure affects number sense and the mental number line in young domestic chicks. *eLife* **14**:RP106356. doi: 10.7554/eLife.106356.

The story of a horse called Clever Hans that was supposed to be able to answer questions about mathematics is one of the most fascinating anecdotes in the history of psychology. When his master, Wilhelm von Osten, asked Hans a maths question, the horse knocked his hoof on the ground until he reached the answer. For example, if the question was “what is five plus three”, Hans would knock his hoof on the ground eight times. It later emerged that von Osten unconsciously gave off subtle cues when the number of knocks equalled the answer, so Hans stopped knocking his hoof (Dehaene, 2011).

Although Hans was not able to do maths, recent studies have shown that some sort of awareness of numbers is widespread among animals that do not speak: examples include newborn infants (Izard et al., 2009), rhesus monkeys (Nieder et al., 2002), crows (Wagener et al., 2018), domestic chicks (Kobylkov et al., 2022) and archerfish (Potrich et al., 2022). Some of these animals only revealed their talent for maths after being trained, but others did so spontaneously. These studies confirm that the ability to handle small discrete numbers is common rather than special. As this talent appears to be innate and biologically rooted, it is considered to be ‘number sense’ (Dehaene, 2011).

In psychology, objects and events in the external world are assumed to be internally represented by a sensory system. Some approaches to the concept of ‘number sense’ assume that numbers are also represented through a sensory system (Figure 1A), but is this assumption valid? Senses are generally modal, and each sensory mode is associated with a particular sense organ (the eyes for vision, and the ears for hearing), with sensory inputs being processed through specialized neural networks for each modality. However, just as there is no sensory system specifically dedicated to time, there is no sensory system dedicated to numbers. Furthermore, numbers are multi-facetted, and a single number can indicate quantity (seven samurai), order (the fourth syllable of ten), or operator (double the reward).

**Figure 1.**
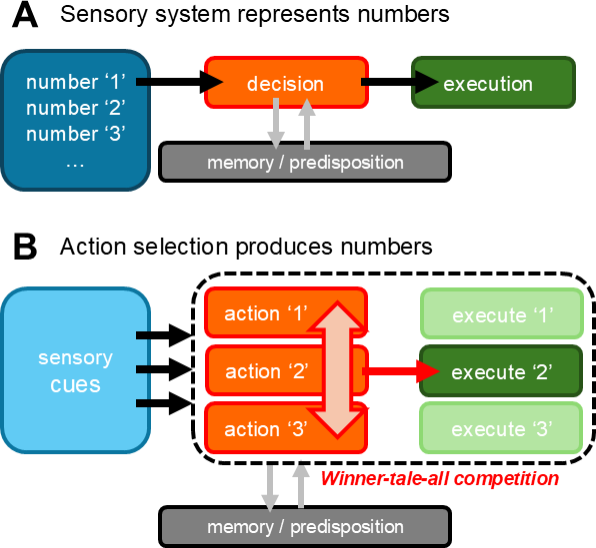
Different models of how the brain processes discrete numbers. (**A**) Schematic diagram of a model in which the sensory system represents numbers. When a decision involving numbers has to be made, the decision process interacts with memory and predisposed biases, but not with the sensory representation of numbers. (**B**) Schematic diagram of a model in which numbers are linked with a decision process in which several discrete actions are competing with each other. When a decision involving numbers has to be made, the decision process interacts with the memory/predisposed biases, a single action ’wins’, and this action is executed, leading to the number-based behavioural execution.

Humans tend to associate small numbers with the left, and large numbers with the right. Some researchers have proposed that this left-to-right ‘mental number line’ is associated with brain lateralization, but this has not been verified. Now, in eLife, Rosa Rugani of the University of Padua and colleagues at Padua and Ohio State University report the results of experiments on young domestic chicks which showed that light-induced brain lateralization was accompanied by the development of the left-to-right mental number line (Rugani et al., 2025).

Rugani et al. studied 100 male domestic chicks: 50 had been incubated in the dark, and 50 were exposed to light between embryonic days 18 and 21. During this period the right eye of the developing chick points outward, while the left points inward, so exposure to light results in an asymmetry in how the two sides of the brain develop. Brain lateralization is actually quite common in animals, but is difficult to manipulate in experiments without using invasive techniques. The fact that light can be used to manipulate brain lateralization in chicks makes them exceptionally useful for such studies.

All the chicks were then trained to peck for a food reward in the fourth container in a line of ten containers arranged along the sagittal axis (that is, the axis that runs from the back of the chick to the front). Finally, the chicks were then made to perform a series of tests. In one test, for example, the line of containers was sometimes rotated 90° clockwise, so that the fourth container became the fourth from the left, and sometimes it was rotated 90° anticlockwise, so that the fourth container became the fourth from the right. The results of these tests suggest that brain lateralization could play an important role in the development of the left-to-right mental number line. We may assume that the light exposure lateralized the sensory representation of numbers (that is, the allocation of numbers to space) during the last phase of embryonic development.

There are, however, other ways to think about numbers. It might be possible to understand number sense as a process that involves selecting one of a number of possible actions, rather than numbers being represented through a sensory system (Figure 1B; Tinbergen, 1951; Cisek, 2007). Although the detailed neural mechanisms involved in this approach remain unclear, neurons in the association cortex (which is part of the cerebral cortex) are reported to code numbers in rhesus monkeys, crows, and chicks (Nieder et al., 2002; Wagener et al., 2018; Kobylkov et al., 2022). The activities of these neurons are tuned to single discrete numbers, as expected from behavioural results. At the level of microcircuits, the neurons coding different numbers are connected via inhibitory interneurons (Wagener et al., 2018), similar to what happens in the song control pathway in sing birds (Mooney and Prather, 2005). If this alternative hypothesis is valid, we will find parallels between action selection and ‘number sense’ in the developmental trajectories of animals when we expose the hypothesis to experimental scrutiny. At the neuronal level, there would be a strong correlation between the activity in certain groups of neurons and the selected action, stronger than that predicted by models in which numbers are represented through a sensory system.

The German mathematician Leopold Kronecker once said: “God created integers, and all others are human artifacts.” Kronecker was attempting to stress the fundamental importance of integers in mathematics, but it is possible that integers are also human artifacts. Furthermore, a subset of integers (small natural numbers) could have preceded humans, first appearing at an early stage in the evolution of vertebrates.
